# *Balamuthia mandrillaris* infection in China: a retrospective report of 28 cases

**DOI:** 10.1080/22221751.2020.1835447

**Published:** 2020-10-28

**Authors:** Lei Wang, Wenjing Cheng, Bing Li, Zhe Jian, Xianlong Qi, Dongjie Sun, Jian Gao, Xuetao Lu, Yi Yang, Kun Lin, Chuanlong Lu, Jiaxi Chen, Chunying Li, Gang Wang, Tianwen Gao

**Affiliations:** Department of Dermatology, Xijing Hospital, Fourth Military Medical University, Xian, People’s Republic of China

**Keywords:** Ameba, *Balamuthia mandrillaris*, cutaneous, encephalitis, free-living ameba, granuloma

## Abstract

*Balamuthia mandrillaris* infection is a rare and fatal disease. We have recorded 28 cases of *Balamuthia mandrillaris* infection during the past 20 years. Eighteen patients (64%) were male and 10 (36%) were female. Patient age ranged from 3 to 74 (mean, 27) years. Patient locations were distributed among 12 Provinces in China. Twenty-seven (96%) patients lived in rural areas, and 17 (61%) patients reported a history of trauma before the appearance of skin lesions. All cases presented with skin lesions as the primary symptom, and 16 (57%) cases developed encephalitis. Histopathology of skin lesions revealed granulomatous changes with histiocytes, lymphocytes, and plasma cells infiltration. Amebas were identified in all cases with immunohistochemical staining. Follow-up information was available in 27 (96%) cases. Fifteen (56%) patients died due to encephalitis and 12 (44%) were free of disease after treatment. Our results show that the clinical characteristics of *Balamuthia mandrillaris* infection in China are very different from those in the US. Infection of traumatized skin may play an important role in the pathogenesis of the disease in China. Encephalitis usually develops 3–4 years after skin lesions in Chinese cases. Patients with only skin lesions have a higher cure rate than patients with encephalitis.

## Introduction

*Balamuthia mandrillaris* was first isolated from a pregnant mandrill baboon that had died from a neurological disease in 1986 [1, 2]. It can infect several animals including gorillas, baboons, gibbons, monkeys [3], horses [4], and dogs [5–7]. *Balamuthia mandrillaris* infection in humans is rare and usually has a fatal outcome. More than 200 cases have been reported worldwide, with most cases reported in the US [8–10] and Peru [11–14]. Some cases have also been reported in the Asia-Pacific region, including Japan [15–19], India [20–22], Thailand [23, 24], Australia [25, 26], and South Korea [27]. Recently, two cases were reported in China [28, 29]. In this study, we describe 28 cases of *Balamuthia mandrillaris* infection collected in our department during the past 20 years.

## Materials and methods

The present study was approved by the Institutional Review Board of Xijing Hospital (KY20202030-C-1). Cases were collected in the Department of Dermatology, Xijing Hospital, Fourth Military Medical University, Xian, China. All cases were diagnosed, treated, and followed up by the principle investigator (Gao TW).

Histological examination of skin lesions was performed in all cases (1-6 biopsies in each patient). Histological examination of brain tissue was available for 4 patients (3 autopsies and 1 excision). Immunohistochemical studies were performed in all patients. The staining was performed on a Ventana Benchmark platform with anti-*Balamuthia mandrillaris* polyclonal antibody (1:1000 dilution, provided by Dr. Kenji Yagita, Tokyo, Japan, who originally obtained it from Dr. Govinda Visvesvara, Centers for Disease Control and Prevention, Atlanta, US). Antigen retrieval was performed with protein K digestion for 30 min.

Polymerase chain reaction was performed with DNA obtained from formalin-fixed, paraffin-embedded tissue of skin lesions from each case. DNA was prepared with the QIAamp kit (No. 56404, Qiagen, Chatsworth, CA). The primer set with an amplicon of 144 bp was designed [30] according to the 16S ribosomal ribonucleic acid gene sequence of *Balamuthia mandrillaris* (Accession number AF477012)-BalF7: 5’-TGATCCAGCAATTTCGCATGT-3’ and BalR150: 5’-TAACACTTGCCCTCTCCGTT-3’. Polymerase chain reaction was performed in a reaction volume of 20 μL, containing 1× Ex Taq buffer, 0.3 units of hot start TaKaRa Ex Taq, 400 μM of each deoxynucleoside triphosphate, 1 μM of each primer, and 2 μL DNA template [31]. Cycling conditions were as follows: 95°C for 10 min, 40 cycles at 95°C/60 s, 55°C/60 s, 72°C/60 s, and 72°C for 10 min.

## Results

### General patient characteristics

**Figure 1.**
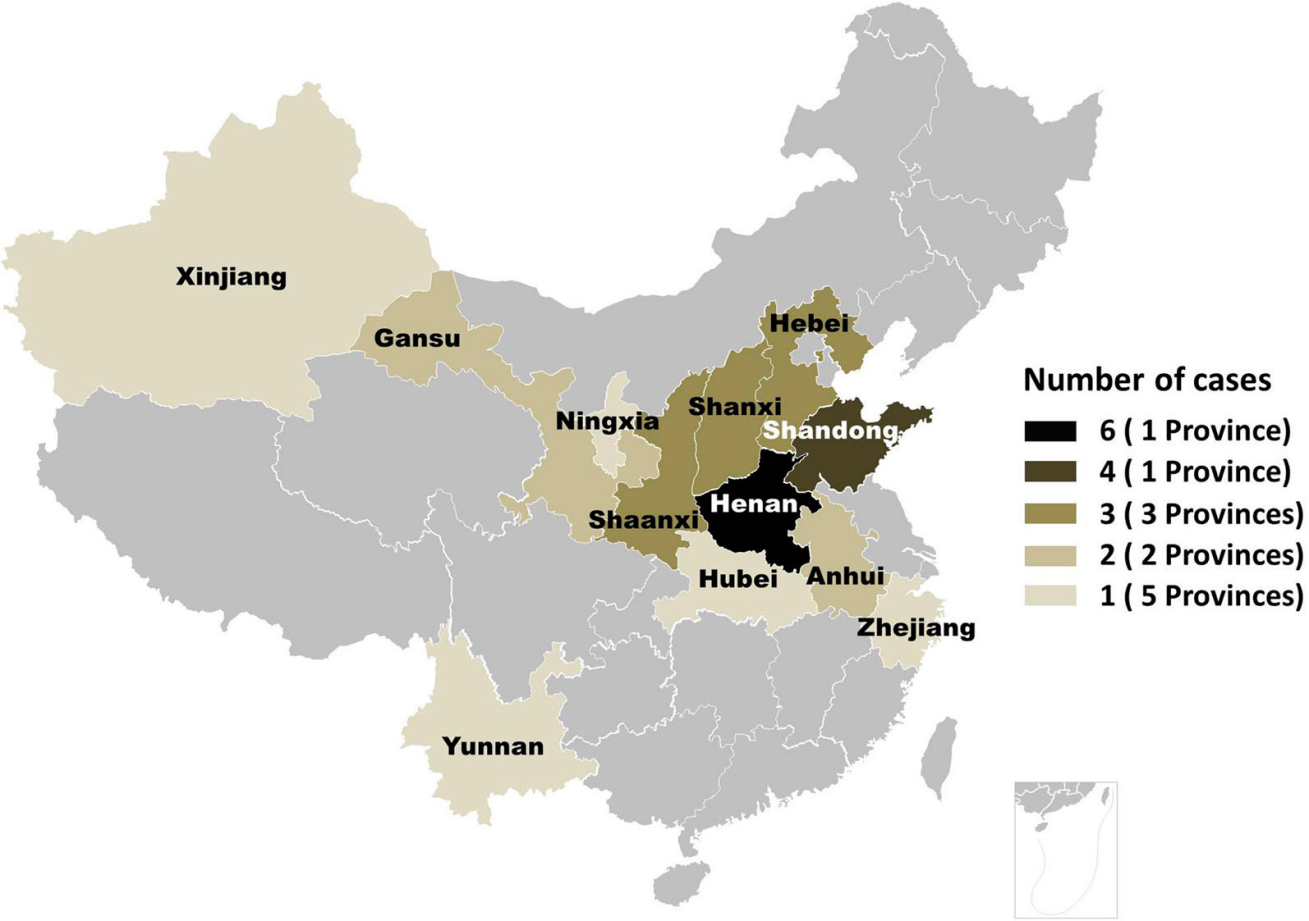
Distribution of 28 cases of *Balamuthia mandrillaris* infection in China.

Eighteen patients (64%) were male and 10 (36%) were female. Patient ages ranged from 3 to 74 years (mean, 27 years); 14 (50%) patients were children (age <18 years). The age at disease onset was 1.5–68 years (mean, 25 years). Their locations were distributed among 12 Provinces of China (Figure 1). Twenty-seven (96%) patients lived in rural areas, and one patient lived in a small city. Seventeen (61%) patients reported a history of local trauma before the appearance of skin lesions, whereas 11 patients reported spontaneous development of the lesions. None of the patients reported immunocompromised conditions such as congenital immunodeficiency, HIV infection, organ transplantation, or autoimmune disease and the use of immunosuppressive drugs (Table 1). General blood findings, including routine blood test, and liver and kidney function, were normal.

**Table 1.** Clinical characteristics, treatments, and outcomes of 28 Chinese patients with cutaneous *Balamuthia mandrillaris* infection.

Cases	Sex/Age (Years)	Causes	Skin symptoms	Brain symptoms	Imaging study with MRI examination	Treatments	Outcomes
1	Male/49	Fall to the ground and traumatized.	Red plaques on right eyebrow, temple and angle of jaw.	No brain symptoms.	Normal.	The skin lesions disappeared after treatment with lincomycin for 5 months. Thereafter, the skin lesions relapsed twice. Finally, the patient was treated with lincomycin for 1 year.	Free of disease for 18 years. The case was described in 2002 [26].
2	Male /6	Traumatized on the temple.	Red plaque on right temple, diameter 7 cm.	Developed encephalitis after 2 years. Presented with fever and headaches.	Infection of the brain stem.	Died before treatment.	Died of disease. Duration between encephalitis and death: 5 days.
3	Female /13	Dog bite on the left face.	Red plaque involving the whole left side of the face.	Developed encephalitis after 5 years. Presented with epilepsy and apathy.	Infection of right temporal lobe, right basal ganglia, bilateral frontal lobes, left hippocampus and hypothalamus.	The skin lesions regressed prominently after treatment with lincomycin and azithromycin, whereas the encephalitis evolved after treatment. Autopsy was performed.	Died of disease. Duration between encephalitis and death: 6 months.
4	Male /8	Unknown.	Red plaque on the left side of the face, diameter 7 cm.	Developed encephalitis after 3 years. Presented with fever, headache, sleepiness, and coma.	Normal during 6-months follow-up. Not performed before death.	Lincomycin, azithromycin, interferon-α, and interleukin-2 were ineffective.	Died of disease. Duration between encephalitis and death: 1 week.
5	Male /5	Unknown.	Red plaque on the nose and right side of the face, diameter 7 cm.	Developed encephalitis after 2 years. Presented with fever and headache.	Multiple infections involving left thalamus, trigone of the left lateral ventricle, left temporal lobe and cerebellum.	Died before treatment.	Died of disease. Duration between encephalitis and death: 1 week.
6	Female /18	Trauma of the face.	Red macule/plaque involving the whole left side of the face and nose.	Developed encephalitis after 9 years. Unable to walk, headache, sleepiness.	Normal during the first consultation. Not performed before death.	The skin lesion regressed after treatment with lincomycin and interferon-γ in the beginning but relapsed after 2 months.	Died of disease. Duration between encephalitis and death: 2 months.
7	Male /20	Injured by a brick.	Red plaque on the left side of the face with focal scales, diameter 9 cm.	Developed encephalitis after 2 years. Presented with apathy, anisocoria, and attenuation of light reflection.	Infections including left cerebellum, right hippocampus, bilateral temporal lobe, left semi oval center, right frontal lobe, and right parietal lobe.	Lincomycin and azithromycin were ineffective. Autopsy was performed.	Died of disease. Duration between encephalitis and death: 1.5 months.
8	Female /7	Traumatized after playing with kids.	Red plaque on the nose and the surrounding skin, diameter 5 cm.	No brain symptoms.	Focal infection of left occipital parietal lobe.	The skin lesion regressed after treatment with lincomycin and interferon-γ, while the brain infection regressed very slowly with the medicine. Thereafter, it was surgically excised and cured.	Free of disease for 13 years.
9	Female /7	Fell to the ground and was traumatized after being knocked down by a car.	Red plaque on the central face, the edge was not clear, diameter larger than 10 cm.	Developed encephalitis after 3 years. Presented with fever and headache.	Normal during the first consultation. Not performed before death.	The skin lesion showed partial regression after treatment with lincomycin and interferon γ for 4 months. After that, the treatment was terminated, and the lesion enlarged again and developed encephalitis.	Died of disease. Duration between encephalitis and death: 1 month.
10	Male /74	Trauma on the face.	Red plaque on the left side of the face, diameter 10 cm.	Developed encephalitis after 3 years. Presented with unsteady walk, tremor, somnolence, and incontinence.	Infection of left frontal lobe.	The skin lesion showed partial regression after treatment with lincomycin and interferon-γ for 2 months. After that, the treatment was terminated, and the lesion enlarged again and developed encephalitis.	Died of disease. Duration between encephalitis and death: 2 months.
11	Male /21	Unknown.	Red plaque on nose and surrounding skin, diameter 7 cm.	Developed encephalitis after 2 years. Presented with headache, somnolence, and coma.	Normal during the first consultation. Not performed before death.	Itraconazole, rifampicin, isoniazid, and ethambutol were ineffective. Lincomycin and interferon-γ were ineffective.	Died of disease. Duration between encephalitis and death: 1 month.
12	Female /61	Traumatized after fall to the ground.	Red plaque on left side of the face, diameter 10 cm.	No brain symptoms.	Normal.	The skin lesion showed regression after treatment with lincomycin, doxycycline, and interferon-γ.	Free of disease for 13 years.
13	Male /3	Traumatized after fall to the ground.	Red plaque on chin, diameter 4.5 cm.	No brain symptoms.	Normal.	The skin lesion was excised and treated with lincomycin and interferon-γ for 6 months.	Free of disease for 12 years.
14	Female /68	Unknown.	Three plaques around a surgical scar. The largest diameter about 2 cm.	No brain symptoms.	Not performed.	The patient underwent surgery but relapsed; hence, the patient was treated with rifampicin, isoniazid, and ethambutol.	Lost to follow-up.
15	Male /13	Trauma of right earlobe.	Plaque on the right ear and the surrounding skin, diameter 8 cm.	Developed encephalitis after 5 years. Presented with sleepiness, reduced speech, and diplopia.	Infection of left parietal, occipital, and frontal lobes.	The skin lesion regressed after treatment with lincomycin, azithromycin and interferon-γ, whereas encephalitis evolved after treatment. Autopsy was performed.	Died of disease. Duration between encephalitis and death: 1 month.
16	Male /5	Unknown.	Red plaque on nose and surrounding skin, diameter of 10 cm.	Developed encephalitis after 3.5 years. Presented with left eye movement restriction, blurred vision, right arm muscle weakness, and inability to stand and walk.	Normal during the first consultation. Not performed before death.	The skin lesion did not regress after treatment with lincomycin, interferon-γ, doxycycline, and rifampin. Azithromycin showed improvement at first but was ineffective after 2 months.	Died of disease. Duration between encephalitis and death: 15 days.
17	Male /39	Fall to the ground when riding a bicycle.	Red plaque on right side of the face and two solitary lesions on arm and waist.	No brain symptoms.	Normal.	The skin lesion was cured after 6 months of treatment with lincomycin and interferon-γ.	Free of disease for 10 years.
18	Male /9	Unknown.	Red plaque on nose, diameter 3 cm.	No brain symptoms.	Normal.	The skin lesion was cured with clindamycin and topical mupirocin for 3 months.	Free of disease for 9 years.
19	Female /22	Nasal congestion after catching a cold.	Red plaque on left nasolabial fold and nose, diameter 4 cm.	Developed encephalitis after 6 months. Presented with headache, dizziness, vomiting and epilepsy.	Infection of left frontal and temporal lobe.	Lincomycin, azithromycin and interferon-α were ineffective.	Died of disease. Duration between encephalitis and death: 2 months.
20	Female /58	Traumatized after fall to the ground.	Dark red plaque on the right ear and the surrounding skin, diameter 8 cm. The helix showed focal absence.	No brain symptoms.	Normal.	The lesion was cured with 1-year treatment with anti-tuberculosis drugs including rifampicin, isoniazid and ethambutol.	Free of disease for 8 years.
21	Male /7	Traumatized after fall to the ground.	Red plaque on the nose and face, diameter 8 cm.	Developed encephalitis after 8 years. Unable to walk, evolved into sleepiness and coma.	Normal during 2-years follow-up. Not performed before death.	The skin lesion showed partial regression after treatment with lincomycin, interferon γ and azithromycin in the beginning, but showed relapse after 3 months.	Died of disease. Duration between encephalitis and death: 2 weeks.
22	Male /48	Traumatized after fall to the ground.	Red plaque on the nose and surrounding skin, diameter 10 cm.	No brain symptoms.	Normal.	The skin lesion was cured after treatment with lincomycin, interferon-γ, and azithromycin for 5 months.	Free of disease for 5 years.
23	Female /4	Traumatized after fall to the ground.	Red plaque on left zygomatic region, diameter 5 cm.	No brain symptoms.	Normal.	The skin lesion was excised, and the patient was treated with lincomycin and interferon-γ.	Free of disease for 5 years.
24	Male /4	Developed after hitting an iron gate.	Red plaque on left side of the face, diameter 12 cm.	Developed encephalitis after 4.5 years. Presenting with sleepiness and coma.	Normal during 2-years follow-up. Not performed before death.	The skin lesion showed partial regression after treatment with lincomycin and interferon-γ but was ineffective after 6 months.	Died of disease. Duration between encephalitis and death: 1 month.
25	Female /57	Unknown.	Red plaque on right side of the face, diameter 10 cm.	No brain symptoms.	Normal.	The skin lesion was cured after treatment with lincomycin and interferon-γ for 3 months.	Free of disease for 4 years.
26	Male /51	Fell to the ground and was traumatized after drinking.	Red plaque on the forehead, diameter 7 cm.	No brain symptoms	Normal.	The skin lesion did not regress with lincomycin, interferon-γ, and azithromycin. Thereafter, it was excised and treated with lincomycin, interferon-γ, and azithromycin for 6 months.	Free of disease for 4 years.
27	Male /15	Unknown.	Red plaque around the nose, diameter 6 cm.	No brain symptoms	Normal.	Skin lesions showed partial regression after treatment with lincomycin, interferon-γ and azithromycin. Six months later it was excised and treated with lincomycin, interferon-γ, and azithromycin for 8 months.	Free of disease for 3 years.
28	Male /69	Unknown.	Red plaque on the forehead, diameter 7 cm.	Developed encephalitis after 4 years. Presenting with sleepiness, coma, and inability to stand and walk.	Normal during the first consultation. Not performed before death.	The skin lesion showed slight regress after treatment with lincomycin and interferon-γ and relapsed after treatment withdrawal.	Died of disease. Duration between encephalitis and death: 2 months.

### Skin manifestation

**Figure 2.**
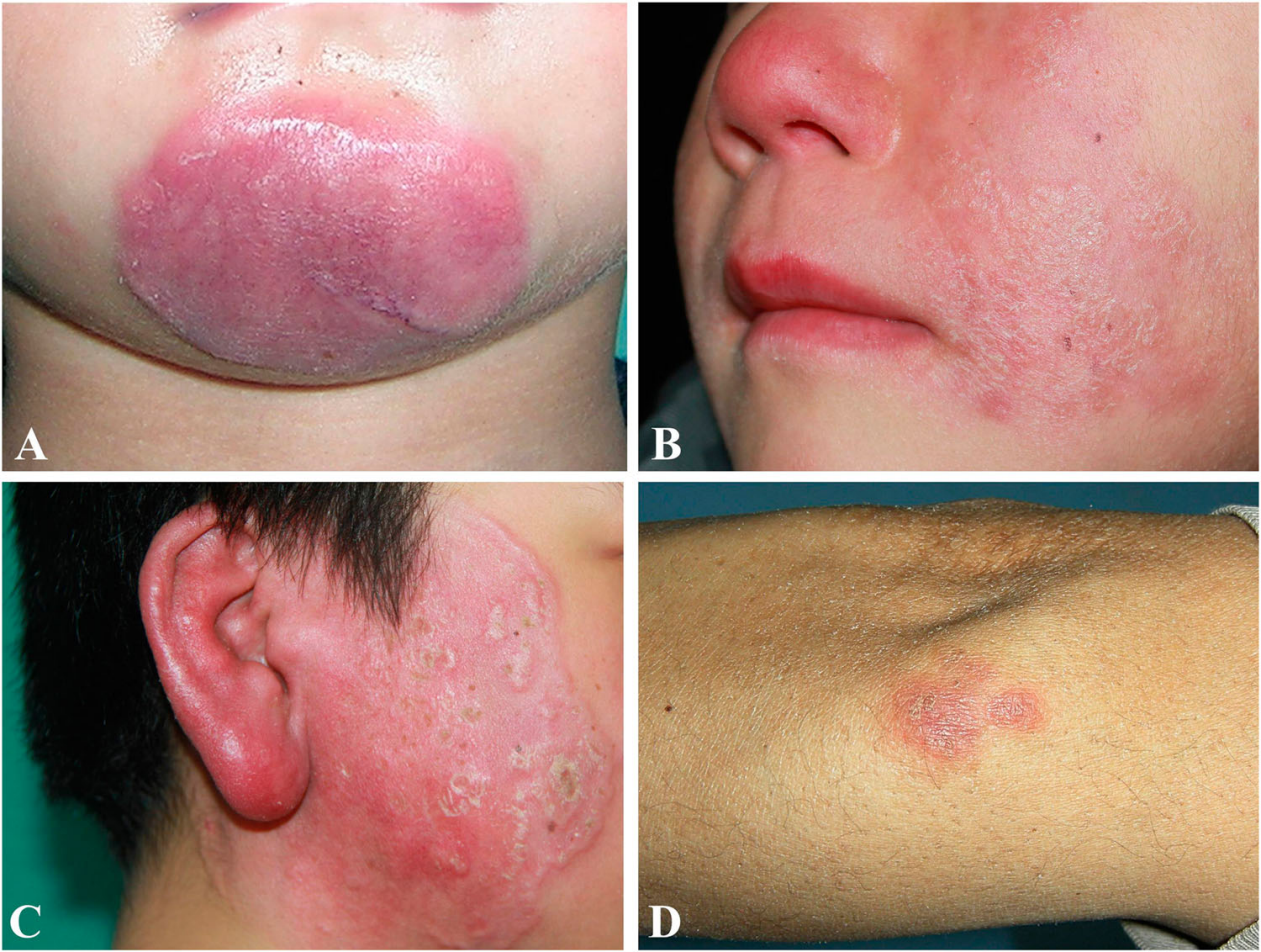
Clinical characteristics of cutaneous *Balamuthia mandrillaris* infection. A-C: Three patients with indurate red plaque on the chin, left face, and right face; D: A patient with plaque on the elbow.

All patients developed skin lesions as the first presentation of the disease. Twenty-six patients showed red plaques distributed on the face (Figure 2(A–C)). One patient showed three separate plaques on the face, arm, and waist, and one patient showed a solitary plaque on the arm (Figure 2(D)). All skin lesions presented as indurate plaques with rubbery to stone hardness. No prominent ulceration or pustule secretion was found in any case.

**Figure 3.**
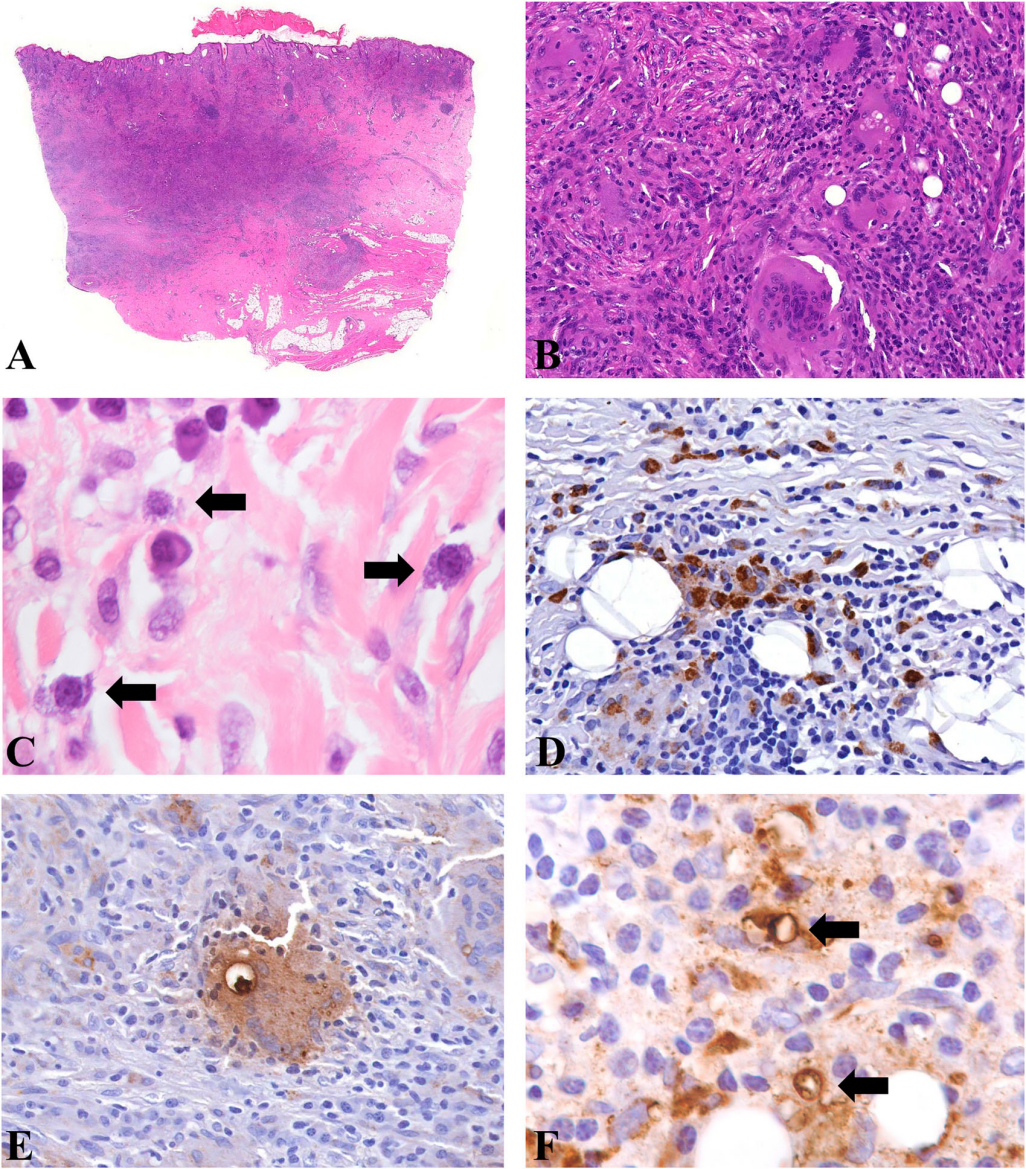
Histopathology of cutaneous *Balamuthia mandrillaris* infection. A, B: Granulomatous infiltration with prominent multinuclear giant cells in the dermis; C: The amebas showed abundant bubbly cytoplasm, round nucleus, and prominent nucleolus, which were easily confused with histiocytes (Arrow). D: Immunohistochemical staining with anti-*Balamuthia* antibody revealed numerous organisms. E: A positively stained ameba engulfed in a multinucleated giant cell. F: Immunohistochemical staining revealed two cysts (arrow).

Histopathology of the skin lesions revealed granulomatous change in the dermis, accompanied by infiltration of histiocytes, multinuclear giant cells, lymphocytes, and plasma cells. Fibrosis of various degrees was also observed (Figure 3(A,B)). At high magnification, the ameba showed morphology similar to histiocytes and was very difficult to identify with hematoxylin and eosin-stained slides (Figure 3(C)); however, immunohistochemical staining revealed a large number of organisms (Figure 3(D–F)). In hematoxylin and eosin-stained skin slides, only trophozoites were identified and their diameters ranged from 10 to 40 µm. In immunohistochemical staining slides, rare cysts with diameters of approximately 10–15 µm were found in 6 skin lesions (Figure 3(F)).

Polymerase chain reaction revealed amplification of the specific 144 bp segment in 27 (96%) cases.

### Encephalic manifestation

**Figure 4.**
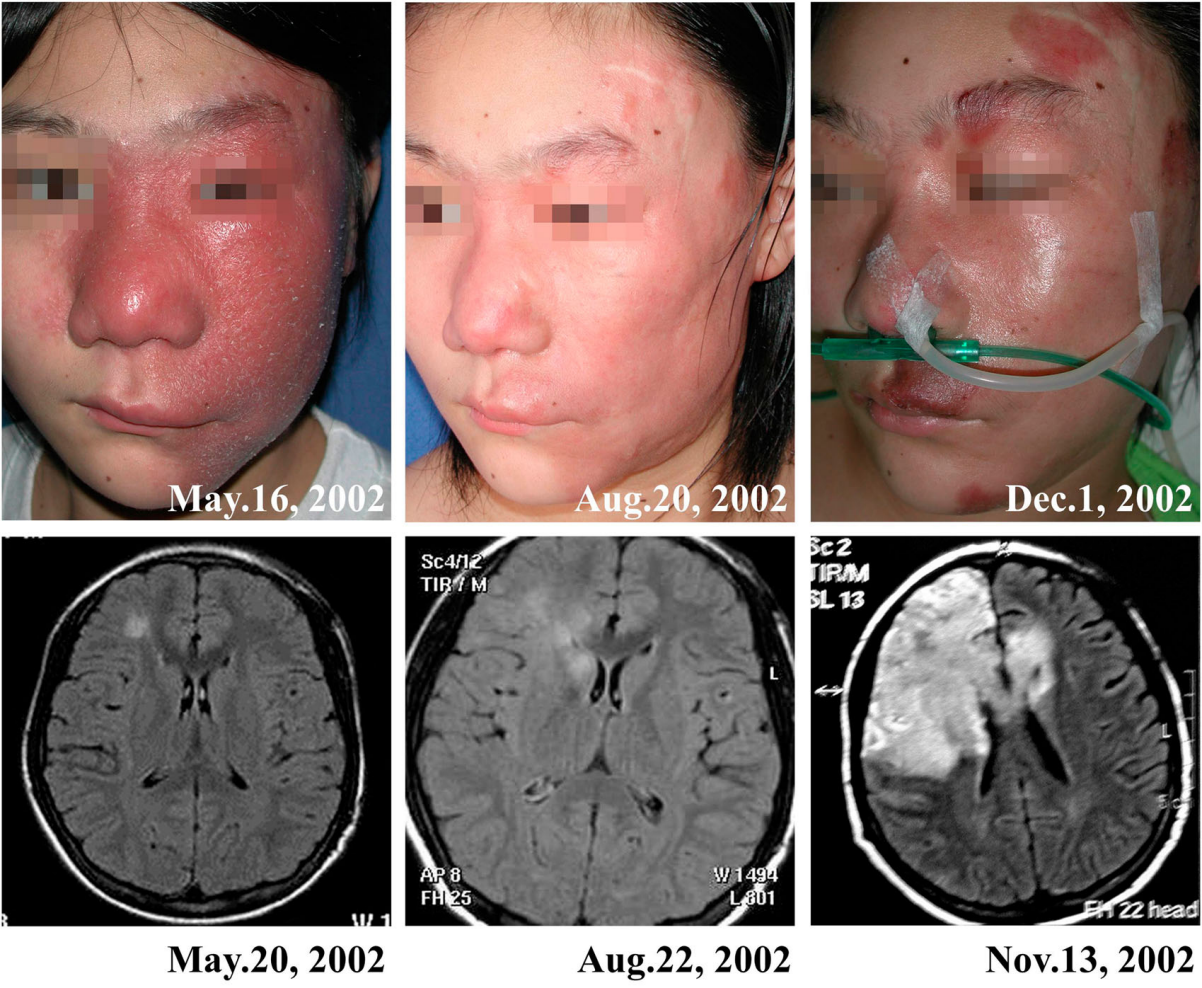
A patient (case 3) showed partial improvement of skin lesions and evolving encephalitis during treatment. A cerebral axial MR fluid-attenuated inversion recovery sequence showed an increase in a few patchy signals in the right frontal lobe in May 2002; however, in November, a large area of increased signal in the right frontotemporal lobe was found. The patient died 6 months after the onset of encephalitis.

**Figure 5.**
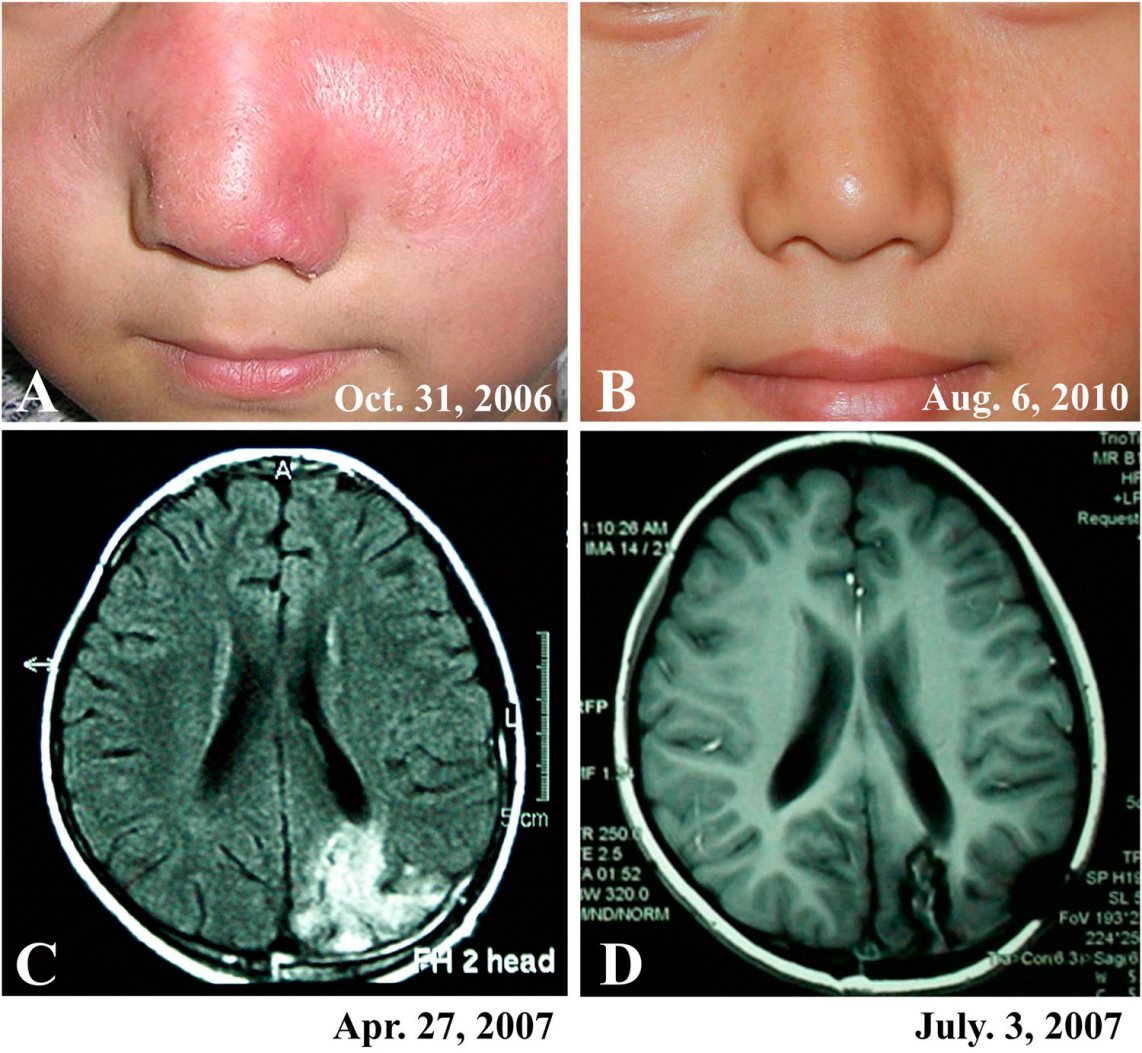
A patient (case 8) with cutaneous and encephalic *Balamuthia mandrillaris* infection survived after treatment with brain surgery and multiple medicines. A: The patient showed red plaque on the face. B: The patient was free of disease at the 4-year follow-up. C: MRI before treatment showed an irregular patchy high signal area in the left occipital lobe. D: Postoperative MRI showed a partial defect of the left occipital bone and partial absence of the left occipital lobe.

Sixteen (57%) patients developed encephalic infection during the course of the disease, of which 8 were verified by imaging studies (Magnetic Resonance Imaging, MRI) and the other eight were verified on the basis of clinical symptoms. Encephalic symptoms included fever, headache, vomiting, sleepiness, epilepsy, changes in consciousness, gait instability, and language disorder, which developed between 0.5 and 9 (mean, 3.7) years after the onset of skin lesions. The MRI examination showed that the encephalitis developed in different brain locations in different patients, which usually showed a low-intensity signal with the T1-weighted image and increased intensity on the T2-weighted image, and associated with edema around the periphery of the lesion (Figures 4 and 5).

**Figure 6.**
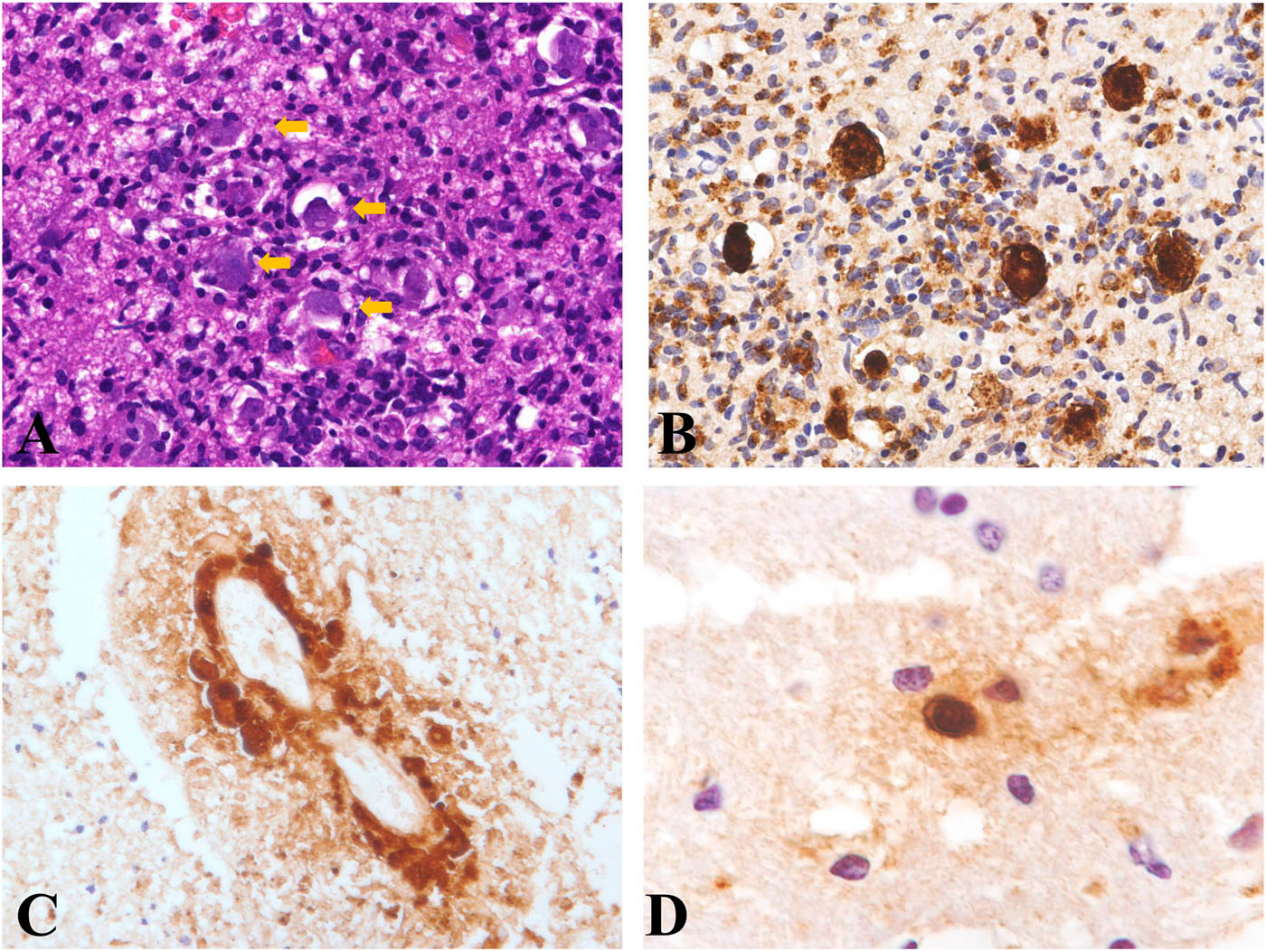
Histopathology of *Balamuthia mandrillaris* encephalitis. A: The amebas were scattered in the brain tissue (arrow). B: Immunohistochemical staining revealed the presence of amebas. C: Immunohistochemical staining revealed a perivascular proliferation of amebas. D: Immunohistochemical staining revealed a cyst in the brain tissue.

Histopathological examination of the brain tissue showed granulomatous changes in brain parenchyma and revealed ameba infiltration at high magnification. The amebas were scattered in the brain tissue and were associated with granulomatous and lymphocytic infiltration. Most of the amebas were trophozoites, with diameters of approximately 10-40 µm (Figure 6). The organism was verified by immunohistochemical staining (Figure 6(B–D)). In some areas, the amebas were distributed around vessels (Figure 6(C)). Cyst was found in one lesion with immunohistochemical staining (Figure 6(D)).

Cerebrospinal fluid examination was performed in three patients with encephalitis and one patient with only skin lesion. Only one patient showed increased lymphocytes in the cerebrospinal fluid, while the other three patients showed normal results.

### Treatments and outcomes

Twenty-seven (96%) patients had follow-up data, and only one patient was lost to follow-up. Fifteen (56%) patients died of encephalic infection; the deaths occurred between 5 days and 6 (mean, 1.4) months after the development of encephalic symptoms. Twelve (44%) patients were free of disease after treatment (Figures 4 and 5). Of the 12 survivors, 11 had only skin lesions, and only one had encephalic infection in a focal area. In this patient, the infected brain tissue was excised, and the patient was concurrently treated with multiple drugs (Figure 5). The most commonly used drugs in our treatment regime include lincomycin, azithromycin, and interferon-γ. However, not all the patients showed good responses to the treatment regime (Table 1).

## Discussion

*Balamuthia mandrillaris* infection is rare and is mainly reported in the North and South Americas, with the US and Peru being the predominant case contributors. This study represents a large case serial of this rare disease in China.

We first noticed the disease in China about 20 years ago. In 2002, we reported 3 cases with skin lesions, of which 2 patients died due to encephalic infection. However, at that time, we were not able to identify the organism and mistakenly thought that they were bacterial infections [32]. In addition to our report, only two cases have been described recently in China [28, 29]. Given the huge population in China, it can be inferred that the disease is exceedingly rare.

The diagnosis of *Balamuthia mandrillaris* infection is still challenging since most dermatologists and neurologists are not familiar with the disease, and cases are rarely encountered in clinical practice. The morphology of *Balamuthia mandrillaris* ameba is very similar to that of histiocytes, which is the primary reason for the disease to be easily misdiagnosed. In our observation, most of the amebas were in the stage of trophozoites, while a small number of cysts were identified using immunohistochemistry (Figure 3(F) and Figure 6(F)). In our experience, granulomatous inflammation with prominent lymphocytes and plasma cell infiltration on the face is suggestive of the disease, and final diagnosis is easily achieved using immunohistochemical methods (Figure 3).

The encephalitis in our cases developed approximately 3–4 years after skin lesions, and the patients died several days to several months after the development of encephalitis. Imaging studies revealed that the encephalitis developed in different locations of brain among different patients, and the results were similar to those observed in the US [10].

Four of our patients underwent verification via histopathological examination of brain tissue. For the other cases with encephalitis, the diagnoses were based on brain symptoms, imaging studies, and verification of amebas in skin lesions. We have to acknowledge that the evidence of *Balamuthia mandrillaris* encephalitis was insufficient in these cases. However, it is likely that the encephalitis in these patients was related with *Balamuthia mandrillaris* infection instead of other causes because encephalitis was the most likely outcome in patients with cutaneous *Balamuthia mandrillaris* infection, if the cutaneous lesions were not cured [10-14]. The routine cerebrospinal fluid examination results in our patients were not suggestive for the diagnosis of ameba encephalitis. In US report, most patients exhibited mildly elevated white blood cell counts with lymphocytic predominance, elevated protein levels, and low-to-normal glucose levels; however, the cerebrospinal fluid profile did not distinguish *Balamuthia mandrillaris* encephalitis from other kinds of encephalitis [10]. According to recent reports from China, two patients were diagnosed using next-generation sequencing of cerebrospinal fluid [28, 29]. Next-generation sequencing of cerebrospinal fluid examination is a non-invasive and fast method to diagnosis amoebic encephalitis; however, the sensitivity and specificity to diagnose early amoebic encephalitis needs to be tested in large case serials.

*Balamuthia mandrillaris* infection has two kinds of clinic presentations. One is represented by cases from the US, in which patients directly develop encephalitis, do not have skin lesions, and usually die within months [8–10]. The other is represented by cases from China and Peru, in which patients show initial skin lesions and develop encephalitis several years later [11–14, 32]. Clinically, cases recorded in China and Peru were less aggressive than those in the US and were easier to diagnose, given that a skin biopsy is easier to perform than a brain biopsy [33]. In addition, patients can also be treated at the stage when only skin symptoms are present, which usually lasts for several years, thereby increasing the chances of successful treatment. Of our 12 surviving patients, 11 had only skin lesions, and only 1 patient had encephalitic infection.

It is not known why most patients in the US develop encephalitis directly, while most patients from China and Peru show initial skin lesions. It is possible that *Balamuthia mandrillaris* ameba is transmitted from polluted soil or water since the organism has been identified and isolated from soil or water from several different locations [34–37]. In our cases, 27 of 28 (96%) patients were from rural areas, and 17 (61%) of them reported a history of trauma. It is reasonable to hypothesize that most of our cases were caused by inoculation of the ameba from contaminated soil. Both China and Peru are developing countries with large rural populations, which are at a higher risk of ameba infection through traumatized skin than the urban population is. However, it is difficult to explain why the lesions most often developed on the patients’ face, since other locations on the body such as the extremities are also easily traumatized.

In our group, about 50% of cases were children, which is similar to cases reported in Peru [11–14] and the US [10]. It is not known why children are prone to this rare infection. In our study, none of the patients were immunocompromised, which differs from observations in the US, where 39% patients were immunocompromised [10].

Treatment of *Balamuthia mandrillaris* remains a challenge. In a study in the US, only 10 of 101 patients (10%) survived the disease [10], whereas in our study, 12 of 27 patients (44%) survived. However, the rather high survival rate was mainly due to the low encephalic infection rates in our group. Only one survivor among our cases developed focal encephalitis during the course of the disease, which was treated by excision of the infected brain tissue (Figure 5). Doyle et al. [25] have reported a case successfully treated using the same method. However, this should not be the first choice in most cases.

We found that lincomycin, azithromycin, interferon-γ, or a combination of these drugs showed effectiveness in treating skin lesions in many cases. We also observed that in some patients, the skin lesions improved gradually, but that the encephalic infection was progressively aggravated (Figure 4). It is possible that the drugs cannot penetrate the blood–brain barrier, and therefore, have poor therapeutic efficiency for encephalitis. Recently, Laurie *et al* [38] screened more than 2000 drugs and identified quinoline nitroxoline as a promising treatment for *Balamuthia mandrillaris* infection. However, currently, there are no reports of cases treated with this drug. Other potential drugs have also been screened *in vitro*; however, the clinical efficacy and side effects of these drugs need to be verified clinically [39, 40].

In conclusion, our results show that *Balamuthia mandrillaris* infection is very rare in China. Patients with *Balamuthia mandrillaris* infection in China have different clinical characteristics and outcomes than those in the US. Dermatologists, pathologists, pediatricians, and neurologists should be alerted to this rare disease when diagnosing unexplained granulomatous lesions.

## References

[1] Visvesvara GS, Martinez AJ, Schuster FL, et al. Leptomyxid ameba, a new agent of amebic meningoencephalitis in humans and animals. J Clin Microbiol. 1990;28:2750–2756. 2280005 10.1128/jcm.28.12.2750-2756.1990 PMC268267

[2] Visvesvara GS, Schuster FL, Martinez AJ. *Balamuthia mandrillaris*, N. G., N. Sp., agent of amebic meningoencephalitis in humans and other animals. J Eukaryot Microbiol. 1993;40:504–514. 8330028 10.1111/j.1550-7408.1993.tb04943.x

[3] Rideout BA, Gardiner CH, Stalis IH, et al. Fatal infections with *Balamuthia mandrillaris* (a free-living amoeba) in gorillas and other old world primates. Vet Pathol. 1997;34:15–22. 9150541 10.1177/030098589703400103

[4] Kinde H, Visvesvara GS, Barr BC, et al. Amebic meningoencephalitis caused by *Balamuthia mandrillaris* (leptomyxid ameba) in a horse. J Vet Diagn Invest. 1998;10:378–381. 9786532 10.1177/104063879801000416

[5] Foreman O, Sykes J, Ball L, et al. Disseminated infection with *Balamuthia mandrillaris* in a dog. Vet Pathol. 2004;41:506–510. 15347823 10.1354/vp.41-5-506

[6] Finnin PJ, Visvesvara GS, Campbell BE, et al. Multifocal *Balamuthia mandrillaris* infection in a dog in Australia. Parasitol Res. 2007;100:423–426. 17033842 10.1007/s00436-006-0302-0

[7] Hodge PJ, Kelers K, Gasser RB, et al. Another case of canine amoebic meningoencephalitis–the challenges of reaching a rapid diagnosis. Parasitol Res. 2011;108:1069–1073. 21161275 10.1007/s00436-010-2197-z

[8] Centers for Disease Control and Prevention (CDC). Balamuthia amebic encephalitis-California, 1999-2007. MMWR Morb Mortal Wkly Rep. 2008;57:768–771. 18636064

[9] Schuster FL, Yagi S, Gavali S, et al. Under the radar: Balamuthia amebic encephalitis. Clin Infect Dis. 2009;48:879–887. 19236272 10.1086/597260

[10] Cope JR, Landa J, Nethercut H, et al. The epidemiology and clinical features of *Balamuthia mandrillaris* disease in the United States, 1974-2016. Clin Infect Dis. 2019;68:1815–1822. 30239654 10.1093/cid/ciy813 PMC7453664

[11] Bravo FG, Alvarez PJ, Gotuzzo E. *Balamuthia mandrillaris* infection of the skin and central nervous system: an emerging disease of concern to many specialties in medicine. Curr Opin Infect Dis. 2011;24:112–117. 21192259 10.1097/QCO.0b013e3283428d1e

[12] Bravo FG, Seas C. *Balamuthia mandrillaris* amoebic encephalitis: an emerging parasitic infection. Curr Infect Dis Rep. 2012;14:391–396. 22729402 10.1007/s11908-012-0266-4

[13] Cabello-Vílchez AM, Rodríguez-Zaragoza S, Piñero J, et al. *Balamuthia mandrillaris* in South America: an emerging potential hidden pathogen in Perú. Exp Parasitol. 2014;145(Suppl):S10–S19. 24858923 10.1016/j.exppara.2014.05.007

[14] Cabello-Vílchez AM. *Balamuthia mandrillaris* in Peru: infection of the skin and central nervous system. In Encephalitis, SM Group. 2016. Available at: https://smjournals.com/ebooks/encephalitis/index.php. [cited 2020 Dec 6].

[15] Shirabe T, Monobe Y, Visvesvara GS. An autopsy case of amebic meningoencephalitis. The first Japanese case caused by *Balamuthia mandrillaris*. Neuropathology. 2002;22:213–217. 12416563 10.1046/j.1440-1789.2002.00444.x

[16] Itoh K, Yagita K, Nozaki T, et al. An autopsy case of *Balamuthia mandrillaris* amoebic encephalitis, a rare emerging infectious disease, with a brief review of the cases reported in Japan. Neuropathology. 2015;35:64–69. 25186798 10.1111/neup.12151

[17] Kobayashi S, Tsukadaira A, Kobayashi S, et al. Amoebic encephalitis in a farmer. Pathology. 2015;47:720–722. 26517637 10.1097/PAT.0000000000000331

[18] Takei K, Toyoshima M, Nakamura M, et al. An Acute case of granulomatous amoebic encephalitis-*Balamuthia mandrillaris* infection. Intern Med. 2018;57:1313–1316. 29321406 10.2169/internalmedicine.0011-17 PMC5980817

[19] Hara T, Yagita K, Sugita Y. Pathogenic free-living amoebic encephalitis in Japan. Neuropathology. 2019;39:251–258. 31243796 10.1111/neup.12582

[20] Prasad K, Bhatia R, Srivastava MV, et al. Fatal subacute necrotising brainstem encephalitis in a young man due to a rareparasitic (Balamuthia) infection. Pract Neurol. 2008;8:112–117. 18344381 10.1136/jnnp.2007.142547

[21] Khurana S, Hallur V, Goyal MK, et al. Emergence of *Balamuthia mandrillaris* meningoencephalitis in India. Indian J Med Microbiol. 2015;33:298–300. 25865989 10.4103/0255-0857.154887

[22] Thamtam VK, Uppin MS, Pyal A, et al. Fatal granulomatous amoebic encephalitis caused by Acanthamoeba in a newly diagnosed patient with systemic lupus erythematosus. Neurol India. 2016;64:101–104. 26755000 10.4103/0028-3886.173662

[23] Krasaelap A, Prechawit S, Chansaenroj J, et al. Fatal Balamuthia amebic encephalitis in a healthy child: a case report with review of survival cases. Korean J Parasitol. 2013;51:335–341. 23864745 10.3347/kjp.2013.51.3.335 PMC3712108

[24] Intalapaporn P, Suankratay C, Shuangshoti S, et al. *Balamuthia mandrillaris* meningoencephalitis: the first case in Southeast Asia. Am J Trop Med Hyg. 2004;70:666–669. 15211011

[25] Doyle JS, Campbell E, Fuller A, et al. *Balamuthia mandrillaris* brain abscess successfully treated with complete surgical excision and prolonged combination antimicrobial therapy. J Neurosurg. 2011;114:458–462. 21073255 10.3171/2010.10.JNS10677

[26] Botterill E, Yip G. A rare survivor of Balamuthia granulomatous encephalitis. Clin Neurol Neurosurg. 2011;113:499–502. 21398024 10.1016/j.clineuro.2011.01.013

[27] Kum SJ, Lee HW, Jung HR, et al. Amoebic encephalitis caused by *Balamuthia mandrillaris*. J Pathol Transl Med. 2019;53:327–331. 31121998 10.4132/jptm.2019.05.14 PMC6755651

[28] Yang Y, Hu X, Min L, et al. *Balamuthia mandrillaris*-related primary amoebic encephalitis in China diagnosed by next generation sequencing and a review of the literature. Lab Med. 2020;51:e20–e26. 31711180 10.1093/labmed/lmz079

[29] Wu X, Yan G, Han S, et al. Diagnosing *Balamuthia mandrillaris* encephalitis via next-generation sequencing in a 13-year-old girl. Emerg Microbes Infect. 2020;9:1379–1387. 32552393 10.1080/22221751.2020.1775130 PMC7473209

[30] Untergasser A, Cutcutache I, Koressaar T, et al. Primer3–new capabilities and interfaces. Nucleic Acids Res. 2012;40: e115. 22730293 10.1093/nar/gks596 PMC3424584

[31] Dietrich D, Uhl B, Sailer V, et al. Improved PCR performance using template DNA from formalin-fixed and paraffin-embedded tissues by overcoming PCR inhibition. PLoS One. 2013;8: e77771. 24155973 10.1371/journal.pone.0077771 PMC3796491

[32] Gao TW, Li CY, Zhao XD, et al. Fatal bacteria granuloma after trauma: a newentity. Br J Dermatol. 2002;147:985–993. 12410712 10.1046/j.1365-2133.2002.04941.x

[33] Guarner J, Bartlett J, Shieh WJ, et al. Histopathologic spectrum and immunohistochemical diagnosis of amebic meningoencephalitis. Mod Pathol. 2007;20:1230–1237. 17932496 10.1038/modpathol.3800973

[34] Schuster FL, Dunnebacke TH, Booton GC, et al. Environmental isolation of *Balamuthia mandrillaris* associated with a case of amebic encephalitis. J Clin Microbiol. 2003;41:3175–3180. 12843060 10.1128/JCM.41.7.3175-3180.2003 PMC165348

[35] Dunnebacke TH, Schuster FL, Yagi S, et al. *Balamuthia mandrillaris* from soil samples. Microbiology. 2004;150:2837–2842. 15347743 10.1099/mic.0.27218-0

[36] Baquero RA, Reyes-Batlle M, Nicola GG, et al. Presence of potentially pathogenic free-living amoebae strainsfrom well water samples in Guinea-Bissau. Pathog Glob Health. 2014;108:206–211. 24934796 10.1179/2047773214Y.0000000143 PMC4069338

[37] Latifi AR, Niyyati M, Lorenzo-Morales J, et al. Presence of *Balamuthia mandrillaris* in hot springs from MazandaranProvince, northern Iran. Epidemiol Infect. 2016;144:2456–2461. 27086943 10.1017/S095026881600073X PMC9150520

[38] Laurie MT, White CV, Retallack H, et al. Functional Assessment of 2,177 U.S. and International drugs Identifies the quinoline nitroxoline as a Potent Amoebicidal Agent against the Pathogen *Balamuthia mandrillaris*. MBio. 2018: e02051–18. 30377287 10.1128/mBio.02051-18 PMC6212833

[39] Rice CA, Troth EV, Russell AC, et al. Discovery of anti-amoebic inhibitors from screening the MMV pandemic response box on *Balamuthia mandrillaris*, Naegleria fowleri, and Acanthamoeba castellanii. Pathogens. 2020 Jun 16;9: 476. 32560115 10.3390/pathogens9060476 PMC7344389

[40] Rice CA, Colon BL, Chen E, et al. Discovery of repurposing drug candidates for the treatment of diseases caused by pathogenic free-living amoebae. PLoS Negl Trop Dis. 2020;14:e0008353. 32970675 10.1371/journal.pntd.0008353 PMC7546510

